# Association between neutrophil percentage-to-albumin ratio and adverse clinical outcomes after successful percutaneous coronary intervention for chronic total occlusion: a cohort study

**DOI:** 10.3389/fimmu.2026.1882018

**Published:** 2026-07-09

**Authors:** Song Wen, Xingjie Huang, Zehan Huang, Yuqing Huang, Hua Yang, Bin Zhang

**Affiliations:** 1 Department of Cardiology, Guangdong Cardiovascular Institute, Guangdong Provincial People’s Hospital, Guangdong Academy of Medical Sciences, Southern Medical University, Guangzhou, Guangdong, China; 2 Department of Cardiology, Fuwai Hospital, National Center for Cardiovascular Diseases, Chinese Academy of Medical Sciences and Peking Union Medical College, Beijing, China; 3 Department of Cardiology, The Second Affiliated Hospital of Guilin Medical University, Guilin, Guangxi, China; 4 Department of Geriatrics, Anning First People’s Hospital Affiliated to Kunming University of Science and Technology, Kunming, Yunnan, China

**Keywords:** chronic total occlusion, inflammation, neutrophil percentage-to-albumin ratio (NPAR), nutrition, outcomes, percutaneous coronary intervention

## Abstract

**Background:**

Chronic total occlusion (CTO) percutaneous coronary intervention (PCI) has improved outcomes, yet residual cardiovascular risk persists. The neutrophil percentage-to-albumin ratio (NPAR), an integrated marker of inflammation and nutritional status, has not been examined in successfully revascularized CTO patients. We investigated whether NPAR is independently associated with long−term adverse outcomes in this population.

**Methods:**

This single−center retrospective cohort study included 1513 consecutive patients who underwent successful CTO PCI. NPAR was calculated as (neutrophil percentage × 100)/albumin (g/dL). The primary endpoint was all−cause mortality, secondary endpoints were cardiovascular mortality and cardiovascular events (cardiovascular death, non−fatal myocardial infarction, non−fatal stroke). Multivariable Cox regression and restricted cubic splines (RCS) assessed the association between NPAR and clinical outcomes. Time-dependent Receiver operating characteristics (ROC) curves were used to evaluate the ability for NPAR to predict all-cause mortality.

**Results:**

During a median follow−up of 810 days, 83 (5.5%) all−cause deaths, 53 (3.5%) cardiovascular deaths, and 73 (4.8%) cardiovascular events occurred. After multivariable adjustment, each 1−standard deviation increase in NPAR was associated with a 50% higher risk of all−cause mortality (HR 1.50, 95% CI 1.23–1.83, P<0.001), a 59% higher risk of cardiovascular mortality (HR 1.59, 95% CI 1.23–2.05, P<0.001), and a 42% higher risk of cardiovascular events (HR 1.42, 95% CI 1.13–1.79, P = 0.003). RCS analysis revealed a linear association between NPAR and all-cause mortality (P for non-linearity = 0.971), cardiovascular mortality (P for non-linearity = 0.150), and major cardiovascular events (P for non-linearity = 0.152). Time-dependent ROC analyses demonstrated that adding NPAR to a basic model comprising age, multi-vessel disease, and LVEF significantly improved discrimination for all-cause mortality at 1, 2, and 3 years (ΔAUC 0.092, 0.076, and 0.058, respectively; all P < 0.0001). The optimal NPAR cut-off values derived from the maximum Youden index were stable across all three time points (15.71, 16.18, and 15.37, respectively), yielding sensitivities of 71.8% to 72.6% and specificities of 73.2% to 75.7%.

**Conclusion:**

In patients undergoing successful CTO PCI, elevated NPAR is independently and linearly associated with increased long-term mortality and major cardiovascular events. This simple, objective biomarker may refine post-intervention risk stratification and identify high-risk individuals warranting intensified secondary prevention.

## Introduction

Chronic total occlusion (CTO) remains one of the most technically challenging lesion subsets in coronary artery disease (CAD) (1, 2). With advances in both equipment and techniques, percutaneous coronary intervention (PCI) for CTO now achieves high success rates in experienced centers, offering meaningful benefits in terms of angina relief, improvement in left ventricular function, and possibly long-term survival (3–5). Nevertheless, even after successful CTO recanalization, a residual risk of major adverse cardiovascular events persists, driven by complex lesion morphology, extensive coronary atherosclerosis, and the underlying systemic inflammatory and metabolic milieu (6, 7). Against this background, there is considerable clinical interest in identifying simple, readily available biomarkers that can refine risk stratification after successful CTO PCI.

Systemic inflammation and nutritional status represent two intertwined pathophysiological axes that critically influence atherosclerotic progression, plaque instability, and cardiovascular outcomes. Neutrophils, as key effector cells of innate immunity, are implicated in oxidative stress, endothelial dysfunction, and pro-thrombotic activity within the coronary circulation (8, 9). Conversely, serum albumin acts as a negative acute-phase reactant and a modulator of systemic antioxidant capacity; hypoalbuminemia reflects not only nutritional depletion but also chronic low-grade inflammation and diminished physiological reserve (10). The neutrophil percentage-to-albumin ratio (NPAR), derived from routine laboratory tests, integrates these two pathways into a single composite metric and has emerged as an independent prognostic biomarker across a range of cardiovascular conditions, including CAD, stroke, acute myocardial infarction, hypertension, atrial fibrillation, heart failure, and chest pain populations (11–21). Its chief advantages are simplicity, objectivity, and minimal additional cost, being calculated from standard complete blood count and chemistry panels. However, no study to date has specifically examined the prognostic value of NPAR in patients who have undergone successful CTO PCI, a distinct cohort characterized by extensive coronary atherosclerosis, frequent multimorbidity, and high procedural complexity, yet with restored vessel patency.

Accordingly, the present cohort study was designed to investigate the association between pre−procedural NPAR and long−term adverse clinical outcomes, including all−cause mortality, cardiovascular mortality, and major cardiovascular events, in a large consecutive series of patients who underwent successful CTO PCI. We hypothesized that higher NPAR levels would be independently associated with an increased risk of these endpoints. By clarifying the role of this integrated inflammatory−nutritional marker, our findings may inform improved risk stratification and guide targeted post−intervention management in this high−risk population.

## Methods

### Study design and population

This single-center retrospective cohort study initially included 1756 consecutive adult patients who underwent PCI for at least one angiographically confirmed CTO lesion at Guangdong Provincial People’s Hospital between February 2011 and December 2023. Patients were excluded if they had procedural failure (n=72), had active systemic infections, autoimmune diseases, active malignancies, end-stage liver disease, or severe hematological disorders at the time of admission (n=0), incomplete NPAR or essential covariate data (n=79), or were lost to follow-up (n=100). After exclusions, 1513 participants were included in the final analysis.

The study protocol complied with the principles of the Declaration of Helsinki and was approved by the Institutional Review Board of Guangdong Provincial People’s Hospital. Written informed consent was obtained from all participants. Reporting followed the Strengthening the Reporting of Observational Studies in Epidemiology (STROBE) guidelines to ensure transparency and methodological rigor.

### Data collection and definitions

CTO PCI was performed in patients with angiographically confirmed CTOs who had refractory symptoms (e.g. angina or dyspnoea) despite optimal medical therapy, objective evidence of viable myocardium, or inducible ischaemia in the CTO territory. Procedural details have been described previously. Coronary angiography and PCI were performed via femoral, radial, or combined access. Crossing strategies, guide catheters, stent types, and the use of intravascular imaging were left to the operator’s discretion. All operators were high−volume operators, each having performed >300 CTO PCI procedures in total, with a minimum of 50 CTO PCIs annually as the primary operator. CTO was defined as complete interruption of antegrade blood flow on coronary angiography, with an estimated occlusion duration of ≥3 months (22). Occlusion duration was determined based on symptom chronology, history of prior myocardial infarction (MI), and previous angiographic studies. Technical success was defined as achievement of Thrombolysis in Myocardial Infarction (TIMI) grade ≥2 antegrade flow in all distal branches ≥2.5 mm with residual stenosis <30% in the target CTO lesion at the end of the procedure. Procedural success was defined as technical success without in−hospital major adverse cardiovascular events (MACE; i.e. death, MI, or clinically driven target vessel revascularization) (22).

Baseline demographic and clinical characteristics were retrospectively collected for all participants. Demographic information included age, sex, comorbidities, smoking status, and history of prior MI or revascularization (PCI or coronary artery bypass grafting [CABG]). Clinical information comprised physical examination findings, laboratory results, imaging data, and discharge medications. Fasting venous blood samples were drawn on the morning of the day prior to or the day of the PCI procedure, after ≥12 hours of overnight fasting. All samples were collected before any procedural intervention, ensuring that NPAR values reflect baseline pre-procedural inflammatory and nutritional status rather than perioperative stress responses. Samples were analyzed at the core laboratory of Guangdong Provincial People’s Hospital under standardized quality control protocols. Measured parameters included complete blood count, total cholesterol (TC), triglycerides (TG), high−density lipoprotein cholesterol (HDL−C), low−density lipoprotein cholesterol (LDL−C), fasting blood glucose (FBG), hemoglobin A1c (HbA1c), C−reactive protein (CRP), blood urea nitrogen (BUN), creatinine (Cr), uric acid (UA), alanine aminotransferase (ALT), aspartate aminotransferase (AST), albumin (ALB), creatine kinase (CK), CK−MB, troponin T (TnT), and N−terminal pro−B−type natriuretic peptide (NT−proBNP). Left ventricular ejection fraction (LVEF) was assessed at rest using the modified biplane Simpson’s rule (23). Diabetes mellitus (DM) was defined by prior diagnosis, use of glucose−lowering agents, or meeting any of the following: FBG ≥7.0 mmol/L, HbA1c ≥6.5%, or 2−hour plasma glucose ≥11.1 mmol/L on oral glucose tolerance testing (24). Hypertension was defined as a prior diagnosis, current antihypertensive treatment, or measured blood pressure ≥140/90 mmHg (25). NPAR was calculated as: (neutrophil percentage of total white blood cell count) × 100/albumin (g/dL) (11–21).

### Follow-up and clinical endpoints

The median follow-up duration was 810 (IQR, 409–1230) days. Follow−up data were obtained through medical record review, outpatient visits, and structured telephone interviews conducted by trained researchers blinded to baseline data. The primary clinical endpoint was defined as all-cause mortality, which was mortality due to any reason. Secondary endpoints were cardiovascular mortality and cardiovascular events, the latter defined as cardiovascular mortality, non−fatal MI, and non−fatal stroke. Cardiovascular mortality was adjudicated unless a clear non−cardiovascular cause was established. Non−fatal MI was defined according to contemporary guidelines as a rise in cardiac troponin accompanied by typical chest pain, serial electrocardiographic changes, identification of intracoronary thrombus on angiography or autopsy, or imaging evidence of new loss of viable myocardium or new regional wall motion abnormality (23). Non−fatal stroke was defined as a new focal neurological deficit lasting >24 hours, confirmed by neuroimaging. All endpoint events were independently verified by two physicians.

### Statistical analysis

Normality of continuous variables was assessed with the Shapiro–Wilk test. Continuous variables are presented as median (interquartile range) and categorical variables as frequency (percentage). Between−group comparisons were performed using the Kruskal–Wallis rank sum test for continuous variables and the chi−square test or Fisher’s exact test for categorical variables.

Kaplan–Meier survival curves with log−rank tests illustrated the incidence of all−cause mortality, cardiovascular mortality, and cardiovascular events across NPAR tertiles. Multivariable Cox proportional hazards models estimated hazard ratios (HRs) and 95% confidence intervals (CIs) for the associations between NPAR and clinical outcomes. Three models were constructed: Model 1 was unadjusted; Model 2 adjusted for age and sex; Model 3 additionally adjusted for smoking, hypertension, DM, dyslipidaemia, prior MI, prior PCI, prior stroke, multi−vessel disease, LDL−C, HDL−C, FBG, HbA1c, creatinine, UA, CRP, LVEF, statin use, and dual antiplatelet therapy. The proportional hazards assumption was verified using Schoenfeld residuals, and no significant violations were detected. Potential dose–response associations between NPAR and clinical outcomes were examined using restricted cubic splines (RCS) with three knots placed at the 30th, 60th, and 90th percentiles.

Discriminatory performance was evaluated using time-dependent receiver operating characteristic analyses to assess the incremental predictive value of NPAR for all-cause mortality at 1, 2, and 3 years of follow-up. A basic clinical model comprising age, multi-vessel disease, and LVEF served as the reference. The area under the curve (AUC) was calculated for both the basic model and the model augmented with NPAR, and the incremental gain was quantified as ΔAUC. The Harrell’s C-statistic was additionally derived from baseline cohort analyses to corroborate incremental discrimination. Optimal cut-off values for NPAR were determined at each respective time point by maximizing the Youden index (sensitivity + specificity - 1).

Exploratory analyses, including interaction testing, assessed the association between NPAR and clinical outcomes across subgroups defined by sex, age (≤60 vs. >60 years), smoking, hypertension, DM, prior MI, LDL-C (<2.6 vs. ≥2.6 mmol/L), and LVEF (<60% vs. ≥60%). Sensitivity analyses were performed to assess the robustness of the primary findings. These included: (1) exclusion of patients with a LVEF <30%; (2) exclusion of patients with a follow-up duration <90 days; (3) exclusion of patients with prior CABG; (4) additional adjustment for LAD as the target vessel; (5) exclusion of patients with a WBC >15 × 10^9^/L or ALB <30 g/L; (6) additional adjustment for ticagrelor and clopidogrel use; and (7) removal of adjustment for CRP.

A two−sided P < 0.05 was considered statistically significant. All analyses were performed using R software, version 4.1.2 (R Foundation for Statistical Computing).

## Results

### Baseline characteristics

The study cohort comprised 1513 participants, with a median age of 59.0 (IQR, 52.0–67.0) years, 1386 (91.6%) were male. Baseline characteristics stratified by NPAR tertiles were summarized in **Table 1**. Compared with patients in the lowest NPAR tertile (T1), those in higher tertiles are older, had a higher SBP, had a higher prevalence of diabetes, prior stroke, multi-vessel disease, but a lower prevalence of prior PCI, and current smoking (all P<0.05). Laboratory profiling revealed that higher NPAR tertiles were characterized by elevated levels of WBC, Neutrophil count, Neutrophil percentage, FBG, HbA1c, Creatinine, BUN, NT−proBNP, TNT, and CRP, alongside lower hemoglobin, lymphocyte count, lymphocyte percentage, HDL, TG, ALT, ALB, CK, and LVEF (all P<0.05). No significant differences were observed across tertiles in the use of statins, β−blockers, dual antiplatelet therapy, calcium channel blockers (CCB), or angiotensin-converting enzyme inhibitor (ACEI)/angiotensin II receptor blocker (ARB) (all P>0.05).

**Table 1:** Baseline characteristics grouped by NPAR levels.

Variables	All (N=1513)	T1 (N = 506)	T2 (N = 503)	T3 (N = 504)	P value
Age, years	59.00 (52.00, 67.00)	55.00 (49.00, 63.00)	59.00 (51.00, 67.00)	63.00 (57.00, 69.00)	<0.001
Male, n (%)	1386 (91.6%)	465 (91.9%)	469 (93.2%)	452 (89.7%)	0.12
SBP, mmHg	130.00 (119.00, 143.00)	127.00 (116.00, 141.00)	130.00 (120.00, 144.00)	132.00 (119.00, 145.00)	0.003
DBP, mmHg	78.00 (70.00, 85.00)	78.00 (71.00, 85.00)	79.00 (70.00, 86.00)	76.00 (69.00, 84.00)	<0.001
Heart rate, beats/min	74.00 (67.00, 82.00)	73.00 (65.00, 81.00)	75.00 (67.00, 82.00)	75.00 (68.00, 83.00)	0.13
Current smoker, n (%)	588 (38.9%)	230 (45.5%)	182 (36.2%)	176 (34.9%)	<0.001
Hypertension, n (%)	902 (59.6%)	287 (56.7%)	299 (59.4%)	316 (62.7%)	0.2
Diabetes, n (%)	602 (39.8%)	184 (36.4%)	187 (37.2%)	231 (45.8%)	0.003
Dyslipidemia, n(%)	423 (28.0%)	144 (28.5%)	157 (31.2%)	122 (24.2%)	0.044
Prior MI, n (%)	237 (15.7%)	80 (15.8%)	66 (13.1%)	91 (18.1%)	0.10
Prior PCI, n (%)	757 (50.0%)	275 (54.3%)	255 (50.7%)	227 (45.0%)	0.012
Prior CABG, n (%)	36 (2.4%)	13 (2.6%)	11 (2.2%)	12 (2.4%)	>0.9
Prior stroke, n (%)	75 (5.0%)	15 (3.0%)	24 (4.8%)	36 (7.2%)	0.009
Laboratory results					
Hb, g/L	135.00 (125.00, 146.00)	140.00 (129.00, 149.00)	137.00 (127.00, 146.00)	130.00 (118.00, 141.00)	<0.001
WBC, 10^9^/L	6.96 (5.84, 8.25)	6.48 (5.63, 7.84)	6.90 (5.78, 7.97)	7.51 (6.27, 8.87)	<0.001
PLT, 10^9^/L	217.00 (180.00, 263.00)	215.00 (182.00, 261.00)	220.00 (183.00, 262.00)	216.50 (174.00, 269.00)	0.7
Neutrophil count, 10^9^/L	4.11 (3.30, 5.16)	3.39 (2.84, 4.11)	4.13 (3.45, 4.99)	5.08 (4.06, 6.18)	<0.001
Neutrophil percentage	0.60 (0.54, 0.66)	0.52 (0.48, 0.56)	0.61 (0.58, 0.64)	0.68 (0.64, 0.72)	<0.001
lymphocyte count, 10^9^/L	1.79 (1.43, 2.22)	2.15 (1.81, 2.64)	1.76 (1.46, 2.15)	1.46 (1.16, 1.79)	<0.001
lymphocyte percentage	0.26 (0.21, 0.32)	0.34 (0.30, 0.38)	0.26 (0.23, 0.29)	0.20 (0.16, 0.24)	<0.001
LDL, mmol/L	2.39 (1.87, 2.95)	2.42 (1.87, 2.95)	2.32 (1.82, 2.86)	2.47 (1.94, 3.01)	0.059
HDL, mmol/L	0.93 (0.82, 1.07)	0.95 (0.83, 1.08)	0.93 (0.83, 1.05)	0.91 (0.79, 1.07)	0.028
TC, mmol/L	3.87 (3.20, 4.60)	3.88 (3.19, 4.60)	3.82 (3.16, 4.54)	3.88 (3.31, 4.65)	0.4
TG, mmol/L	1.47 (1.06, 2.09)	1.63 (1.16, 2.30)	1.47 (1.07, 2.16)	1.38 (0.99, 1.89)	<0.001
FBG, mmol/L	5.50 (4.85, 6.90)	5.28 (4.79, 6.53)	5.49 (4.82, 6.65)	5.73 (5.00, 7.60)	<0.001
HbA1c, %	6.10 (5.70, 6.80)	6.10 (5.70, 6.70)	6.00 (5.60, 6.60)	6.20 (5.70, 6.98)	0.002
Creatinine, µmol/L	84.00 (72.90, 100.19)	79.90 (71.22, 91.00)	84.19 (72.90, 99.34)	90.18 (74.80, 111.82)	<0.001
BUN, µmol/L	5.59 (4.52, 6.83)	5.36 (4.42, 6.57)	5.60 (4.70, 6.63)	5.90 (4.50, 7.95)	<0.001
UA, μmol/L	404.90 (337.00, 478.00)	401.22 (344.70, 479.70)	413.30 (343.00, 480.70)	395.97 (325.10, 470.65)	0.11
ALT, U/L	22.00 (16.00, 32.00)	25.00 (18.00, 34.00)	22.00 (15.00, 31.00)	20.50 (14.00, 30.00)	<0.001
AST, U/L	22.00 (18.00, 27.00)	22.00 (19.00, 27.00)	21.00 (17.00, 26.00)	21.00 (17.00, 27.00)	0.072
ALB, g/L	38.80 (36.56, 41.00)	40.70 (38.60, 42.70)	39.20 (37.47, 40.73)	36.41 (34.00, 38.29)	<0.001
NT-proBNP, pg/ml	229.40 (73.40, 749.70)	129.80 (47.30, 377.00)	175.10 (73.00, 604.10)	602.10 (170.10, 1,841.00)	<0.001
TNT, pg/ml	17.20 (9.70, 41.24)	12.05 (8.40, 24.60)	17.00 (10.40, 31.70)	29.20 (13.80, 94.55)	<0.001
CK, U/L	87.00 (63.00, 122.00)	89.00 (70.00, 125.00)	86.00 (64.00, 116.00)	81.00 (56.50, 125.00)	0.002
CK-MB, U/L	10.20 (10.00, 13.00)	10.10 (10.00, 13.00)	10.10 (10.00, 13.00)	10.50 (10.00, 13.10)	>0.9
CRP, mg/L	1.97 (0.50, 6.20)	0.92 (0.50, 3.18)	1.70 (0.50, 4.40)	5.49 (1.10, 17.13)	<0.001
LVEF, %	58.00 (46.00, 63.00)	60.00 (53.00, 64.00)	59.00 (48.00, 64.00)	54.00 (40.00, 62.00)	<0.001
NPAR	15.56 (13.84, 17.44)	13.19 (11.93, 13.84)	15.58 (14.95, 16.13)	18.37 (17.44, 20.19)	<0.001
Angiographic characteristics					
Target CTO artery					0.6
RCA, n (%)	858 (56.7%)	279 (55.1%)	293 (58.3%)	286 (56.7%)	
LAD, n (%)	713 (47.1%)	238 (47.0%)	238 (47.3%)	223 (47.0%)	
LCX, n (%)	199 (13.2%)	76 (15.2%)	64 (12.7%)	59 (11.7%)	
LM, n (%)	3 (0.2%)	1 (0.2%)	1 (0.2%)	1 (0.2%)	
Multi-vessel disease, n(%)	1354 (89.5%)	439 (86.8%)	461 (91.7%)	454 (90.1%)	0.035
Medications					
DAPT, n(%)	1477 (97.6%)	499 (98.6%)	489 (97.2%)	489 (97.0%)	0.2
Statin, n(%)	1494 (98.7%)	503 (99.4%)	496 (98.6%)	495 (98.2%)	0.2
β‐Blocker, n(%)	1211 (80.0%)	415 (82.0%)	395 (78.5%)	401 (79.6%)	0.4
CCB, n(%)	318 (21.0%)	97 (19.2%)	108 (21.5%)	113 (22.4%)	0.4
ACEI/ARB, n(%)	902 (59.7%)	303 (60.0%)	302 (60.0%)	297 (58.9%)	>0.9
SGLT2i, n(%)	272 (18.0%)	105 (20.8%)	85 (16.9%)	82 (16.3%)	0.13
Clopidogrel, n(%)	910 (60.1%)	261 (51.6%)	315 (62.6%)	334 (66.3%)	<0.001
Ticagrelor, n(%)	592 (39.1%)	242 (47.8%)	185 (36.8%)	165 (32.7%)	<0.001
All-cause mortality, n(%)	83 (5.5%)	8 (1.6%)	20 (4.0%)	55 (10.9%)	<0.001
Cardiovascular mortality, n(%)	53 (3.5%)	5 (1.0%)	14 (2.8%)	34 (6.7%)	<0.001
Cardiovascular events, n(%)	73 (4.8%)	7 (1.4%)	23 (4.6%)	43 (8.5%)	<0.001

NPAR, neutrophil percentage-to-albumin ratio; LVEF, center ventricular ejection fraction; PCI, percutaneous coronary intervention; CABG, coronary artery bypass grafting; SBP, systolic blood pressure; DBP, diastolic blood pressure; MI, myocardial infarction; TC, total cholesterol; HDL-C, high-density lipoprotein cholesterol; LDL-C, low-density lipoprotein cholesterol; TG, triglycerides; FBG, fasting blood glucose; CRP, C-reactive protein; BUN, blood urea nitrogen; UA, uric acid; HbA1c, hemoglobin A1c; Hb, hemoglobin; WBC, white blood cell; PLT, platelet; ALT, Alanine Aminotransferase; BNP, brain natriuretic peptide; TNT, Troponin T; CK, creatine kinase; CK-MB, creatine kinase-MB; AST, aspartate aminotransferase; ALB, albumin; RCA, right coronary artery; LAD, Left Anterior descending artery; LCX, Left circumflex artery; LM, Left main coronary artery; DAPT, dual antiplatelet therapy; CCB, calcium channel blocker; ACEI, angiotensin-converting enzyme inhibitor; ARB, angiotensin II receptor blocker; SGLT2i, sodium-glucose cotransporter-2 inhibitors.

### Association of NPAR with clinical outcomes in patients with successful CTO PCI

**Figure 1:**
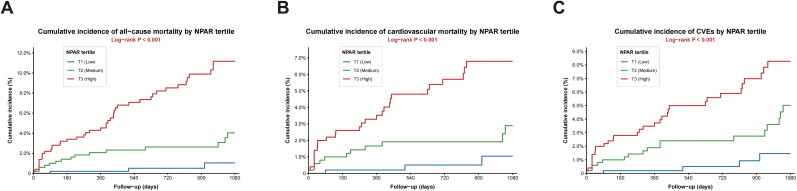
Kaplan-Meier curves of the cumulative incidence of **(A)** all-cause mortality, **(B)** cardiovascular mortality, and **(C)** cardiovascular events according to NPAR.

**Figure 2:**
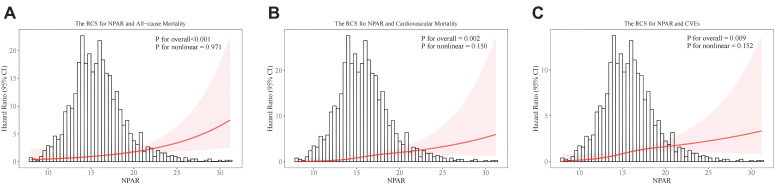
RCS analysis of the NPAR for incidence of **(A)** all−cause mortality, **(B)** cardiovascular mortality, and **(C)** cardiovascular events.

During a median follow-up of 810 (IQR, 409-1230) days, 83 (5.5%) all-cause deaths, 53 (3.5%) cardiovascular deaths, and 73 (4.8%) major cardiovascular events were recorded. Kaplan-Meier curves demonstrated a graded separation of cumulative incidence for all three endpoints across NPAR tertiles (**Figure 1**). In the unadjusted model (Model 1), each 1-standard deviation (SD) increase in NPAR was associated with a 90% higher risk of all-cause mortality (HR 1.90, 95% CI 1.63-2.20, P < 0.001), an 83% higher risk of cardiovascular mortality (HR 1.83, 95% CI 1.51-2.22, P < 0.001), and a 66% higher risk of cardiovascular events (HR 1.66, 95% CI 1.39-1.98, P < 0.001). After comprehensive multivariable adjustment (Model 3), each 1-SD increase in NPAR remained significantly associated with a 50% higher risk of all-cause mortality (HR 1.50, 95% CI 1.23-1.83, P < 0.001), a 59% higher risk of cardiovascular mortality (HR 1.59, 95% CI 1.23-2.05, P < 0.001), and a 42% higher risk of cardiovascular events (HR 1.42, 95% CI 1.13-1.79, P = 0.003). When NPAR was analyzed by tertiles, a graded association was observed. Compared with the lowest tertile (T1), patients in the highest tertile (T3) had a 2.73-fold higher risk of all-cause mortality (HR 2.73, 95% CI 1.24-5.99, P = 0.013), a 3.37-fold higher risk of cardiovascular mortality (HR 3.37, 95% CI 1.24-9.16, P = 0.017), and a 3.31-fold higher risk of cardiovascular events (HR 3.31, 95% CI 1.33-7.30, P = 0.009) (**Table 2**). RCS analysis revealed a linear association between NPAR and all-cause mortality (P for non-linearity = 0.971), cardiovascular mortality (P for non-linearity = 0.150), and major cardiovascular events (P for non-linearity = 0.152) (**Figure 2**).

**Table 2:** Associations between NPAR and clinical outcomes in participants after successful CTO PCI.

Outcomes	Model 1		Model 2		Model 3	
	HR (95%CI)	P-value	HR (95%CI)	P-value	HR (95%CI)	P-value
All-cause mortality						
Per 1-SD increase in NPAR	1.90 (1.63-2.20)	<0.001	1.77 (1.51-2.08)	<0.001	1.50 (1.23-1.83)	<0.0001
T1	Reference		Reference		Reference	
T2	2.29 (1.01-5.21)	0.048	1.98 (0.87-4.51)	0.105	1.49 (0.64-3.47)	0.351
T3	6.01 (2.86-12.63)	<0.001	4.34 (2.04-9.24)	<0.001	2.73 (1.24-5.99)	0.013
Cardiovascular mortality						
Per 1-SD increase in NPAR	1.83 (1.51-2.22)	<0.001	1.69 (1.38-2.08)	<0.001	1.59 (1.23-2.05)	<0.001
T1	Reference		Reference		Reference	
T2	2.63 (0.95-7.32)	0.063	2.34 (0.84-6.53)	0.104	2.05 (0.71-5.88)	0.184
T3	6.12 (2.39-15.66)	<0.001	4.55 (1.75-11.85)	0.002	3.37 (1.24-9.16)	0.017
Cardiovascular events						
Per 1-SD increase in NPAR	1.66 (1.39-1.98)	<0.001	1.52 (1.26-1.84)	<0.001	1.42 (1.13-1.79)	0.003
T1	Reference		Reference		Reference	
T2	3.06 (1.31-7.13)	0.010	2.65 (1.13-6.20)	0.025	2.24 (0.93-5.38)	0.071
T3	5.22 (2.34-11.62)	<0.001	3.77 (1.67-8.51)	0.001	3.31 (1.33-7.30)	0.009

Model 1 adjust for: None.

Model 2 adjust for: age and sex.

Model 3 adjust for: age, sex, smoking, hypertension, DM, dyslipidaemia, prior MI, prior PCI, prior stroke, multi−vessel disease, LDL−C, HDL−C, FBG, HbA1c, creatinine, UA, CRP, LVEF, statin use, and dual antiplatelet therapy.

HR, hazard ratio; CI, confidence interval; NPAR, neutrophil percentage-to-albumin ratio; CTO, chronic total occlusion; PCI, percutaneous coronary intervention.

### Predictive value of NPAR for all-cause mortality in patients with successful CTO PCI

**Figure 3:**
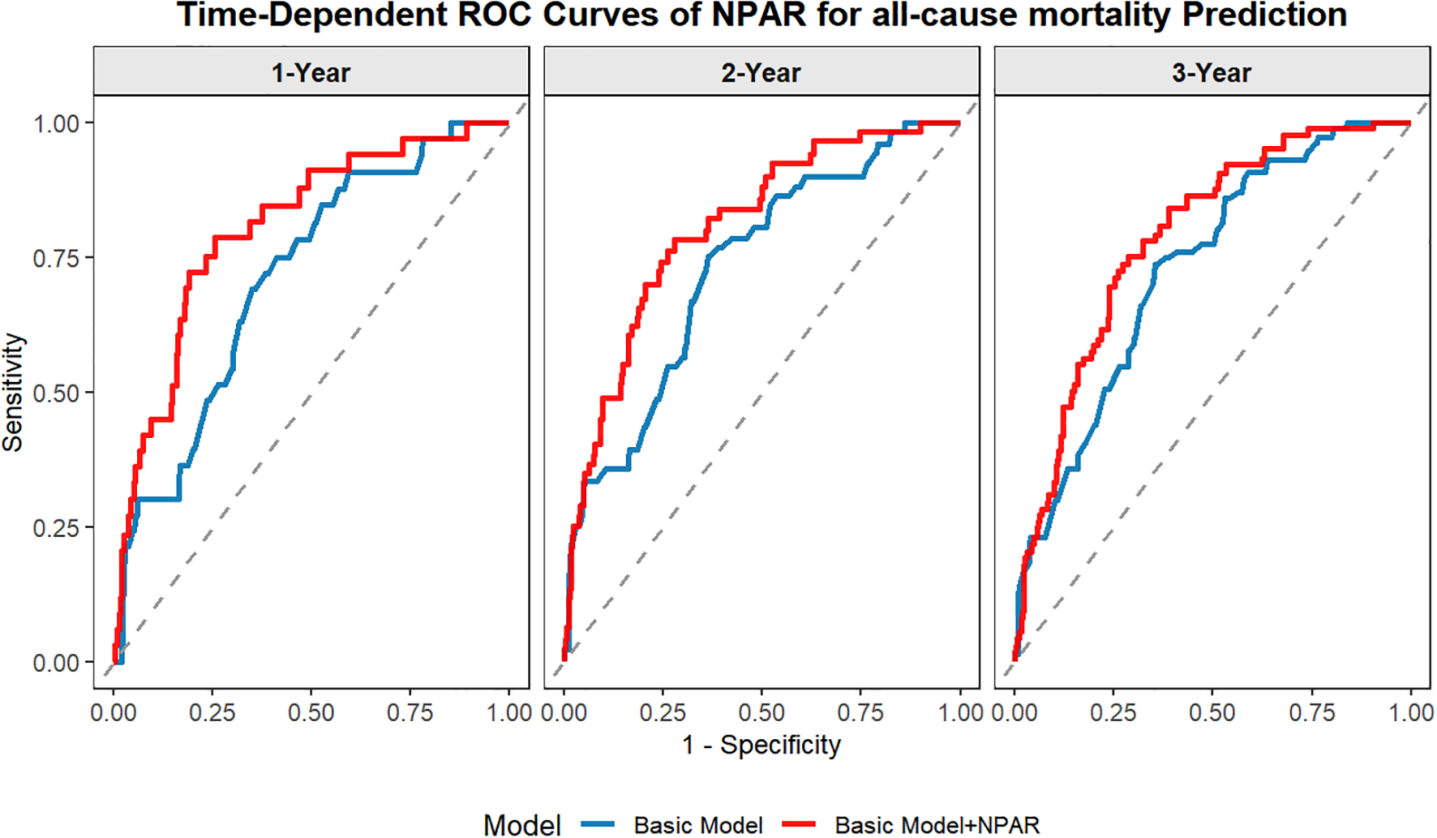
Time-dependent receiver operating characteristic curves of NPAR for all-cause mortality prediction. Basic model included: age, multi-vessel disease, and LVEF.

Time-dependent receiver operating characteristic analyses revealed that the addition of NPAR to the basic risk model, comprising age, multi-vessel disease, and LVEF, substantially improved the discriminatory capacity for all-cause mortality at 1, 2, and 3 years of follow-up (**Table 3**; **Figure 3**). Specifically, the area under the curve (AUC) increased from 0.712 to 0.804 at 1 year (ΔAUC = 0.092, P < 0.0001), from 0.729 to 0.805 at 2 years (ΔAUC = 0.076, P < 0.0001), and from 0.728 to 0.786 at 3 years (ΔAUC = 0.058, P < 0.0001) (**Table 3**). Notably, the most pronounced incremental gain was observed at the 1-year landmark, suggesting that NPAR may confer particular prognostic value for early post-procedural risk stratification. This incremental predictive value was further corroborated by the C-statistics derived from the baseline cohort analyses. The basic model exhibited a C-statistic of 0.717 (95% CI: 0.662–0.773), which significantly increased to 0.789 (95% CI: 0.736–0.839) upon the incorporation of NPAR, yielding a ΔC-statistic of 0.072 (95% CI: 0.031–0.120; P < 0.0001) (**Supplementary Table 1**). This finding robustly confirms that NPAR provides independent prognostic information beyond that of conventional risk factors. Furthermore, the optimal cut-off values for NPAR remained consistently stable across the three time points—15.71 at 1 year (Youden index: 0.4829), 16.18 at 2 years (Youden index: 0.4655), and 15.37 at 3 years (Youden index: 0.4540). These thresholds demonstrated moderate, yet well-balanced, diagnostic performance, with sensitivities and specificities ranging from 71.77% to 72.60% and 73.23% to 75.70%, respectively (**Supplementary Table 2**).

**Table 3:** Predictive performance of NPAR for all-cause mortality across time points.

Time point	AUC (95%CI)		ΔAUC (95%CI)	P-value
	Basic model	Basic model + NPAR		
1-year	0.712 (0.629-0.794)	0.804 (0.730-0.878)	0.092 (0.037-0.159)	<0.0001
2-year	0.729 (0.659-0.799)	0.805 (0.745-0.865)	0.076 (0.033-0.130)	<0.0001
3-year	0.728 (0.666-0.789)	0.786 (0.731-0.841)	0.058 (0.019-0.113)	<0.0001

Basic model included: age, multi-vessel disease, and LVEF.

### Subgroup analyses

Stratified analyses were performed to evaluate the association between NPAR and all-cause mortality across various subgroups (**Table 4**). The association remained directionally consistent across all evaluated strata. No significant interactions were observed (all P for interaction > 0.05), suggesting that the relationship between NPAR and all-cause mortality is robust and not materially modified by baseline characteristics.

**Table 4:** Subgroup analysis of association of NPAR and all-cause mortality in participants with successful CTO PCI.

Subgroups		All-cause mortality	N	HR (95% CI)	P value	P for interaction
Sex	Male	75	1,386	1.51 (1.22-1.87)	<0.001	0.922
	Female	8	127	11.98 (6.22-23.09)	<0.001	
Age	≤60	26	814	1.40 (0.99-1.96)	0.054	0.186
	>60	57	699	1.80 (1.35-2.39)	<0.001	
Current Smoking	Yes	28	588	1.73 (1.21-2.48)	0.003	0.528
	No	55	925	1.54 (1.19-1.99)	<0.001	
Hypertension	Yes	62	902	1.46 (1.13-1.90)	0.004	0.155
	No	21	611	1.80 (1.32-2.44)	<0.001	
Diabetes	Yes	44	602	1.38 (1.01-1.89)	0.042	0.193
	No	39	911	1.88 (1.38-2.56)	<0.001	
Prior MI	Yes	21	237	1.52 (0.91-2.52)	0.108	0.446
	No	62	1,276	1.47 (1.17-1.85)	0.001	
LDL-C	≥2.6	33	590	1.46 (1.02-2.08)	0.036	0.755
	<2.6	50	923	1.44 (1.13-1.82)	0.003	
LVEF	<60	63	817	1.54 (1.23-1.92)	<0.001	0.610
	≥60	20	696	1.39 (0.75-2.56)	0.296	

Models adjust for age, sex, smoking, hypertension, DM, dyslipidaemia, prior MI, prior PCI, prior stroke, multi−vessel disease, LDL−C, HDL−C, FBG, HbA1c, creatinine, UA, CRP, LVEF, statin use, and dual antiplatelet therapy.

HR, hazard ratio; CI, confidence interval; NPAR, neutrophil percentage-to-albumin ratio; CTO, chronic total occlusion; LVEF, center ventricular ejection fraction; PCI, percutaneous coronary intervention; MI, myocardial infarction; LDL-C, low-density lipoprotein cholesterol.

### Sensitivity analyses

Multiple sensitivity analyses confirmed the robustness of the primary findings (**Supplementary Tables 3–S9**. After sequentially excluding patients with a follow-up duration <90 days, those with prior CABG, those with a LVEF <30%, those with a WBC >15 × 10^9^/L or ALB <30 g/L, and after additionally adjusting for LAD as the target vessel, ticagrelor use, clopidogrel use, and removal of adjustment for CRP, the positive associations between NPAR and clinical outcomes remained statistically significant and materially unchanged. Across all sensitivity models, each 1-SD increase in NPAR remained significantly associated with all-cause mortality (HRs 1.45–1.51, all P ≤ 0.010), cardiovascular mortality (HRs 1.43–1.74, all P ≤ 0.015), and major adverse cardiovascular events (HRs 1.30–1.55, all P ≤ 0.040), supporting the reliability of the primary findings.

## Discussion

In this large single-center cohort of 1513 patients who underwent successful CTO PCI, we report three principal findings. First, NPAR, an integrated biomarker of systemic inflammation and nutritional status, was independently associated with long-term all-cause mortality, cardiovascular mortality, and major cardiovascular events after comprehensive multivariable adjustment. Second, the association followed a linear dose–response pattern without evidence of non-linearity. Third, the addition of NPAR to a basic clinical model comprising age, multi-vessel disease, and LVEF significantly improved risk discrimination at 1, 2, and 3 years of follow-up (ΔAUC 0.092, 0.076, and 0.058, respectively; all P < 0.0001), with optimal cut-off values derived from the maximum Youden index remaining stable across all time points (15.71, 16.18, and 15.37, respectively). These associations remained robust across multiple sensitivity analyses and consistent across key subgroups, suggesting that NPAR may serve as a simple, readily available biomarker for risk stratification after successful CTO PCI.

To our knowledge, this is the first study to evaluate the prognostic value of NPAR specifically in patients with successful CTO PCI. Our findings extend the growing body of evidence linking NPAR to adverse outcomes in broader cardiovascular populations. In a study of 18,469 hypertensive adults from the National Health and Nutrition Examination Survey (NHANES) during a median follow-up of 105 months, higher NPAR significantly increased the risks of all-cause (HR 1.80, 95% CI 1.54-2.12) and cardiovascular mortality (HR 1.54, 95% CI 1.24-1.91) (13). Wang et al. reported that NPAR ≥17.326 was an independent prognostic risk factor in patients with ACS undergoing PCI (HR 3.01, 95% CI 1.42-6.41) (16). Among 6846 community-dwelling adults with chest pain, each unit increase in NPAR was associated with an 11% higher risk of all-cause mortality and a 14% increased risk of cardiovascular mortality (19). In 935 participants with CAD, those in the highest NPAR tertile had significantly elevated risks of all-cause mortality (HR 1.76, 95% CI 1.19-2.61) and cardiovascular mortality (HR 3.12, 95% CI 1.67-5.81) compared with the lowest tertile (21). Furthermore, a retrospective cohort study of 622 patients with chronic heart failure found that higher NPAR was independently associated with increased risks of 90−day (highest vs. lowest tertile: HR 2.21, 95% CI 1.01-4.86), 1−year (HR 2.13, 95% CI 1.30-3.49), and 2−year all−cause mortality (HR 2.06, 95% CI 1.37-3.09) (18). The present study now fills an important gap by demonstrating that this relationship holds true in the specific context of successfully revascularized CTO patients—a group characterized by extensive coronary atherosclerosis, a high prevalence of comorbidities such as diabetes and multi−vessel disease, and a residual risk of ischemic events despite successful recanalization. Notably, the magnitude of association we observed is comparable to or slightly higher than that reported for other inflammatory biomarkers in post-PCI populations, such as high-sensitivity CRP or the neutrophil-to-lymphocyte ratio. However, NPAR offers the practical advantage of being derived from routine laboratory panels without additional cost or turnaround time, making it attractive for clinical practice.

The biological plausibility of our findings rests on the dual pathophysiological pathways integrated within NPAR. Neutrophils are central effectors of innate immunity in atherosclerosis. Beyond acute plaque destabilization, they contribute to chronic endothelial dysfunction, oxidative stress, and microvascular injury through the release of reactive oxygen species, proteolytic enzymes, and neutrophil extracellular traps (9, 26–28). In the CTO milieu, where chronic ischemia and hibernating myocardium coexist, sustained neutrophil activation may perpetuate adverse myocardial remodeling and impair microvascular recovery even after antegrade flow has been restored (29). Conversely, serum albumin functions as a negative acute-phase reactant with potent antioxidant and anti-inflammatory properties; hypoalbuminemia reflects not merely hepatic synthetic impairment and nutritional deficiency but also the cumulative burden of chronic inflammation and frailty (10, 30, 31). The ratio of these two parameters thus captures a pathophysiological equilibrium: when neutrophil percentage rises and albumin falls, the net effect is a pro-inflammatory, catabolic state with diminished physiological reserve. The linear relationship observed in RCS analyses further supports the notion that this risk is incrementally conferred across the entire spectrum of NPAR values, without an obvious threshold effect, implying that even modest elevations in inflammatory-nutritional imbalance may translate into measurable increases in long-term cardiovascular risk.

It is also noteworthy that the association between NPAR and clinical outcomes remained significant after adjustment for CRP, a well-established inflammatory marker. This suggests that NPAR may capture complementary biological information, perhaps reflecting more chronic, bone-marrow-derived inflammatory activity and nutritional status rather than acute-phase reactant dynamics alone. Whether NPAR merely mirrors existing disease severity or actively contributes to disease progression remains to be elucidated; nevertheless, its independence from CRP in multivariable models supports its potential additive value in risk stratification. Patients with higher NPAR in our cohort indeed exhibited a more adverse baseline risk profile: they were older, had higher systolic blood pressure, a greater prevalence of DM, prior stroke, and multi-vessel disease, and displayed worse metabolic parameters and lower LVEF. Although we carefully adjusted for these factors, NPAR may additionally capture residual inflammatory and nutritional risk not fully accounted for by traditional covariates.

Subgroup analyses demonstrated that the association between NPAR and all-cause mortality remained consistent across most clinical strata in patients with successful CTO PCI. The marked male predominance (91.6%) reflects the well-documented sex imbalance in CTO PCI cohorts globally, wherein women are less frequently referred for invasive management of complex coronary disease. Nevertheless, this disproportion raises concerns regarding generalizability to female patients. No significant interaction was observed by sex (P for interaction = 0.922). Although female patients exhibited a numerically higher hazard ratio (HR 11.98, 95% CI 6.22–23.09) compared with male patients (HR 1.51, 95% CI 1.22–1.87), this apparent difference was likely driven by the small female sample size, as reflected by the wide confidence interval. Accordingly, the sex-specific estimates should be interpreted with caution, and the data support a broadly similar prognostic effect of NPAR across both sexes. The NPAR–mortality association remained directionally consistent and statistically significant across most other subgroups, and no significant interactions were detected for age, smoking status, hypertension, diabetes, prior MI, LDL-C, or LVEF. These findings suggest that NPAR, as a composite marker of inflammation and nutritional status, provides robust prognostic information independent of traditional cardiovascular risk profiles. In conclusion, NPAR is consistently associated with all-cause mortality across clinically relevant subgroups following successful CTO PCI. The absence of significant interactions indicates that NPAR is a broadly applicable prognostic biomarker in this population, including in both men and women, although larger cohorts of women are needed to confirm the precise effect size in female patients.

From a translational perspective, the simplicity and objectivity of NPAR constitute its most compelling clinical advantage. Calculated from the standard complete blood count and chemistry panel, it requires no additional cost, specialized equipment, or procedural delay. In the context of successful CTO PCI, where patients are already subject to intensive pharmacological management and surveillance, a pre-procedural NPAR assessment could facilitate risk-adaptive post-intervention strategies. For example, patients with elevated NPAR may warrant more aggressive lipid-lowering therapy, tighter glycaemic control, or early referral to cardiac rehabilitation programs that address nutritional status and systemic inflammation. While NPAR was independently associated with outcomes after comprehensive adjustment, our study was not designed to assess its incremental prognostic value beyond existing risk models (e.g., SYNTAX score, GRACE risk score). Future studies should formally evaluate whether adding NPAR to established risk scores improves reclassification and calibration before recommending its routine clinical use. Additionally, we performed time-dependent ROC analyses and determined the optimal NPAR cut-off values for all-cause mortality using the maximum Youden index. The identified thresholds were remarkably stable across the 1-, 2-, and 3-year landmarks (15.71, 16.18, and 15.37, respectively), converging around a clinically actionable threshold of approximately 15.4–16.2. At these cut-offs, NPAR demonstrated moderate yet well-balanced discriminatory performance, with sensitivities of 71.8% to 72.6% and specificities of 73.2% to 75.7%. These data suggest that an NPAR value exceeding ~15.5–16.2 may serve as a practical screening tool for identifying high-risk patients during the early post-procedural period. Nevertheless, because dichotomization of continuous biomarkers sacrifices statistical information, we recommend that this threshold be used as an initial risk-stratification aid rather than a definitive decision boundary. External validation in independent CTO PCI cohorts is warranted before this cut-off can be integrated into routine clinical practice.

The major strengths of this study include the relatively large sample size of consecutive patients with successful CTO PCI, rigorous adjudication of clinical endpoints by two independent physicians blinded to baseline data, and comprehensive adjustment for a wide range of potential confounders. The long follow-up duration and low rate of loss to follow-up enhance the reliability of outcome estimates. Furthermore, comprehensive subgroup and sensitivity analyses yielded consistent results. Several limitations warrant consideration. First, our analysis was restricted to patients who underwent successful CTO PCI, excluding those with procedural failure. While this design allowed us to investigate the prognostic value of NPAR in a homogeneously revascularized cohort, it introduces selection bias and limits extrapolation to patients in whom revascularization was unsuccessful or not attempted. Second, the study population was predominantly male (91.6%), which reflects the well-documented sex disparity in CTO PCI cohorts. Whether our findings apply equally to women remains uncertain, and future studies with larger female samples are needed to validate the prognostic role of NPAR in this under-represented subgroup. Second, despite extensive covariate adjustment, residual confounding from unmeasured factors such as dietary habits, physical activity, and genetic predisposition cannot be ruled out. And our study did not capture several angiographic and procedural variables known may to influence CTO PCI outcomes, including J-CTO score, retrograde crossing strategy, use of intravascular imaging (IVUS/OCT), total stent length, contrast volume, and peri-procedural complications. Although we adjusted for multi-vessel disease and target vessel location, unmeasured confounding by these lesion- and procedure-specific factors cannot be excluded. Future prospective studies should incorporate these variables to better establish the independent prognostic value of NPAR. Third, NPAR was assessed from a single pre-procedural blood sample; we were unable to evaluate whether temporal changes in NPAR during follow-up would refine prognostic accuracy or reflect treatment response. Fourth, our study spanned a 12-year period, during which CTO PCI techniques, procedural success, operator experience, and secondary prevention strategies have evolved substantially. These temporal changes may have influenced both procedural outcomes and long-term event rates. Although we adjusted for medication use at discharge, we could not fully account for changes in practice patterns over time. Future studies should examine whether the prognostic value of NPAR remains consistent across different eras of CTO PCI. Finally, because this is an observational study, causality cannot be established; randomized controlled trials or Mendelian randomization studies would be required to determine whether lowering NPAR (e.g., through nutritional support or anti-inflammatory therapy) improves outcomes.

## Conclusion

In patients undergoing successful CTO PCI, elevated NPAR is independently and linearly associated with increased risks of all-cause mortality, cardiovascular mortality, and cardiovascular events. This simple, low-cost composite marker of inflammation and nutritional status may help refine risk stratification and identify high-risk individuals who could benefit from targeted secondary prevention strategies. External validation in larger, multicenter prospective cohorts is warranted before routine clinical application.

## Data Availability

The raw data supporting the conclusions of this article will be made available by the authors, without undue reservation.
