# Supplementary material for: Association between neutrophil percentage-to-albumin ratio and adverse clinical outcomes after successful percutaneous coronary intervention for chronic total occlusion: a cohort study
Source: Front Immunol. 2026 Jul 9;17:1882018. doi: 10.3389/fimmu.2026.1882018 (PMC13391297; doi:10.3389/fimmu.2026.1882018)
Supplement: Supplementary file 1 [file Table1.docx]

Table S1. Incremental predictive value of NPAR.

| **Models** | **C statistics** |  | **ΔC statistics** |  |
| --- | --- | --- | --- | --- |
|  | **Estimate(95%CI)** |  | **Estimate(95%CI)** | ***P*-value** |
| Basic model | 0.717 (0.662-0.773) |  | Reference |  |
| Basic model + NPAR | 0.789 (0.736-0.839) |  | 0.072 (0.031-0.120) | <0.0001 |

Basic model included: age, multi-vessel disease, and LVEF.

Table S2. Optimal cut-off value of NPAR for predicting the all-cause mortality in participants with successful CTO PCI.

| **Time point** | **Cut-off value** |  | **Youden index** |  | **Sensitivity** |  | **Specificity** |
| --- | --- | --- | --- | --- | --- | --- | --- |
| 1-year | 15.71 |  | 0.4829 |  | 72.60% |  | 75.70% |
| 2-year | 16.18 |  | 0.4655 |  | 71.77% |  | 74.78% |
| 3-year | 15.37 |  | 0.4540 |  | 72.18% |  | 73.23% |

Table S3. Associations between NPAR and clinical outcomes in participants with successful CTO PCI after excluded participants with LVEF＜30% (N = 1434)

|  |  | **All-cause mortality** | |  | **Cardiovascular mortality** | |  | **Cardiovascular events** | |
| --- | --- | --- | --- | --- | --- | --- | --- | --- | --- |
|  |  | **HR (95%CI)** | ***P*-value** |  | **HR (95%CI)** | ***P*-value** |  | **HR (95%CI)** | ***P*-value** |
| Per 1-SD increase in NPAR |  | 1.45 (1.16-1.80) | <0.001 |  | 1.43 (1.07-1.90) | 0.015 |  | 1.30 (1.02-1.67) | 0.037 |
| T1 |  | Reference |  |  | Reference |  |  | Reference |  |
| T2 |  | 1.80 (0.70-4.65) | 0.224 |  | 3.43 (0.94-12.45) | 0.061 |  | 3.00 (1.11-8.16) | 0.031 |
| T3 |  | 2.89 (1.17-7.12) | 0.021 |  | 4.15 (1.18-14.64) | 0.027 |  | 3.46 (1.28-9.36) | 0.014 |

Models adjust for: age, sex, smoking, hypertension, DM, dyslipidaemia, prior MI, prior PCI, prior stroke, multi-vessel disease, LDL-C, HDL-C, FBG, HbA1c, creatinine, UA, CRP, LVEF, statin use, and dual antiplatelet therapy.

HR, hazard ratio; CI, confidence interval; NPAR, neutrophil percentage-to-albumin ratio; CTO, chronic total occlusion; PCI, percutaneous coronary intervention.

Table S4. Associations between NPAR and clinical outcomes in participants with successful CTO PCI after excluded participants with prior CABG (N = 1477)

|  |  | **All-cause mortality** | |  | **Cardiovascular mortality** | |  | **Cardiovascular events** | |
| --- | --- | --- | --- | --- | --- | --- | --- | --- | --- |
|  |  | **HR (95%CI)** | ***P*-value** |  | **HR (95%CI)** | ***P*-value** |  | **HR (95%CI)** | ***P*-value** |
| Per 1-SD increase in NPAR |  | 1.50 (1.24-1.82) | <0.001 |  | 1.59 (1.24-2.03) | <0.001 |  | 1.43 (1.15-1.79) | 0.002 |
| T1 |  | Reference |  |  | Reference |  |  | Reference |  |
| T2 |  | 1.46 (0.63-3.39) | 0.382 |  | 1.95 (0.68-5.64) | 0.216 |  | 2.23 (0.92-5.36) | 0.074 |
| T3 |  | 2.77 (1.26-6.09) | 0.012 |  | 3.32 (1.22-9.05) | 0.019 |  | 3.16 (1.34-7.44) | 0.008 |

Models adjust for: age, sex, smoking, hypertension, DM, dyslipidaemia, prior MI, prior PCI, prior stroke, multi-vessel disease, LDL-C, HDL-C, FBG, HbA1c, creatinine, UA, CRP, LVEF, statin use, and dual antiplatelet therapy.

HR, hazard ratio; CI, confidence interval; NPAR, neutrophil percentage-to-albumin ratio; CTO, chronic total occlusion; PCI, percutaneous coronary intervention.

Table S5. Associations between NPAR and clinical outcomes in participants with successful CTO PCI after excluded participants with follow-up≤90 day (N = 1477)

|  |  | **All-cause mortality** | |  | **Cardiovascular mortality** | |  | **Cardiovascular events** | |
| --- | --- | --- | --- | --- | --- | --- | --- | --- | --- |
|  |  | **HR (95%CI)** | ***P*-value** |  | **HR (95%CI)** | ***P*-value** |  | **HR (95%CI)** | ***P*-value** |
| Per 1-SD increase in NPAR |  | 1.51 (1.19-1.90) | <0.001 |  | 1.63 (1.17-2.27) | 0.004 |  | 1.34 (1.01-1.77) | 0.040 |
| T1 |  | Reference |  |  | Reference |  |  | Reference |  |
| T2 |  | 1.08 (0.42-2.73) | 0.876 |  | 1.42 (0.41-4.85) | 0.580 |  | 1.91 (0.73-5.02) | 0.189 |
| T3 |  | 2.47 (1.05-5.80) | 0.038 |  | 3.22 (1.04-10.03) | 0.043 |  | 2.93 (1.14-7.50) | 0.025 |

Models adjust for: age, sex, smoking, hypertension, DM, dyslipidaemia, prior MI, prior PCI, prior stroke, multi-vessel disease, LDL-C, HDL-C, FBG, HbA1c, creatinine, UA, CRP, LVEF, statin use, and dual antiplatelet therapy.

HR, hazard ratio; CI, confidence interval; NPAR, neutrophil percentage-to-albumin ratio; CTO, chronic total occlusion; PCI, percutaneous coronary intervention.

Table S6. Associations between NPAR and clinical outcomes in participants with successful CTO PCI additionally adjusted for LAD (N = 1513).

|  |  | **All-cause mortality** | |  | **Cardiovascular mortality** | |  | **Cardiovascular events** | |
| --- | --- | --- | --- | --- | --- | --- | --- | --- | --- |
|  |  | **HR (95%CI)** | ***P*-value** |  | **HR (95%CI)** | ***P*-value** |  | **HR (95%CI)** | ***P*-value** |
| Per 1-SD increase in NPAR |  | 1.51 (1.24-1.84) | <0.001 |  | 1.59 (1.23-2.06) | <0.001 |  | 1.42 (1.12-1.79) | 0.003 |
| T1 |  | Reference |  |  | Reference |  |  | Reference |  |
| T2 |  | 1.47 (0.63-3.41) | 0.375 |  | 2.02 (0.70-5.82) | 0.191 |  | 2.27 (0.95-5.45) | 0.066 |
| T3 |  | 2.73 (1.24-6.00) | 0.013 |  | 3.37 (1.24-9.16) | 0.017 |  | 3.13 (1.33-7.35) | 0.009 |

Models adjust for: age, sex, smoking, hypertension, DM, dyslipidaemia, prior MI, prior PCI, prior stroke, multi-vessel disease, LDL-C, HDL-C, FBG, HbA1c, creatinine, UA, LVEF, LAD, statin use, and dual antiplatelet therapy.

HR, hazard ratio; CI, confidence interval; NPAR, neutrophil percentage-to-albumin ratio; CTO, chronic total occlusion; LAD, left Anterior descending artery; PCI, percutaneous coronary intervention.

Table S7. Associations between NPAR and clinical outcomes in participants with successful CTO PCI after excluded patients with WBC＞15 or ALB ＜30 (N = 1466).

|  |  | **All-cause mortality** | |  | **Cardiovascular mortality** | |  | **Cardiovascular events** | |
| --- | --- | --- | --- | --- | --- | --- | --- | --- | --- |
|  |  | **HR (95%CI)** | ***P*-value** |  | **HR (95%CI)** | ***P*-value** |  | **HR (95%CI)** | ***P*-value** |
| Per 1-SD increase in NPAR |  | 1.45 (1.09-1.93) | 0.010 |  | 1.74 (1.23-2.47) | 0.002 |  | 1.55 (1.15-2.09) | 0.004 |
| T1 |  | Reference |  |  | Reference |  |  | Reference |  |
| T2 |  | 1.42 (0.58-3.48) | 0.445 |  | 2.29 (0.73-7.18) | 0.156 |  | 2.47 (0.98-6.24) | 0.057 |
| T3 |  | 2.48 (1.06-5.77) | 0.036 |  | 3.33 (1.10-10.10) | 0.033 |  | 3.04 (1.22-7.58) | 0.017 |

Models adjust for: age, sex, smoking, hypertension, DM, dyslipidaemia, prior MI, prior PCI, prior stroke, multi-vessel disease, LDL-C, HDL-C, FBG, HbA1c, creatinine, UA, LVEF, LAD, statin use, and dual antiplatelet therapy.

HR, hazard ratio; CI, confidence interval; NPAR, neutrophil percentage-to-albumin ratio; CTO, chronic total occlusion; PCI, percutaneous coronary intervention.

Table S8. Associations between NPAR and clinical outcomes in participants with successful CTO PCI additionally adjusted for Ticagrelor, and Clopidogrel (N = 1513).

|  |  | **All-cause mortality** | |  | **Cardiovascular mortality** | |  | **Cardiovascular events** | |
| --- | --- | --- | --- | --- | --- | --- | --- | --- | --- |
|  |  | **HR (95%CI)** | ***P*-value** |  | **HR (95%CI)** | ***P*-value** |  | **HR (95%CI)** | ***P*-value** |
| Per 1-SD increase in NPAR |  | 1.46 (1.20-1.77) | <0.001 |  | 1.53 (1.20-1.94) | <0.001 |  | 1.40 (1.13-1.75) | 0.003 |
| T1 |  | Reference |  |  | Reference |  |  | Reference |  |
| T2 |  | 1.33 (0.57-3.12) | 0.510 |  | 1.80 (0.61-5.28) | 0.286 |  | 2.06 (0.85-4.99) | 0.109 |
| T3 |  | 2.56 (1.16-5.64) | 0.020 |  | 3.41 (1.24-9.32) | 0.017 |  | 3.11 (1.32-7.31) | 0.009 |

Models adjust for: age, sex, smoking, hypertension, DM, dyslipidaemia, prior MI, prior PCI, prior stroke, multi-vessel disease, LDL-C, HDL-C, FBG, HbA1c, creatinine, UA, LVEF, statin use, dual antiplatelet therapy, Ticagrelor, and Clopidogrel.

HR, hazard ratio; CI, confidence interval; NPAR, neutrophil percentage-to-albumin ratio; CTO, chronic total occlusion; PCI, percutaneous coronary intervention.

Table S9. Associations between NPAR and clinical outcomes in participants with successful CTO PCI excluded adjusting for CRP (N = 1513).

|  |  | **All-cause mortality** | |  | **Cardiovascular mortality** | |  | **Cardiovascular events** | |
| --- | --- | --- | --- | --- | --- | --- | --- | --- | --- |
|  |  | **HR (95%CI)** | ***P*-value** |  | **HR (95%CI)** | ***P*-value** |  | **HR (95%CI)** | ***P*-value** |
| Per 1-SD increase in NPAR |  | 1.47 (1.24-1.76) | <0.001 |  | 1.48 (1.19-1.84) | <0.001 |  | 1.40 (1.15-1.71) | <0.001 |
| T1 |  | Reference |  |  | Reference |  |  | Reference |  |
| T2 |  | 1.50 (0.65-3.48) | 0.346 |  | 2.04 (0.71-5.86) | 0.185 |  | 2.27 (0.94-5.44) | 0.067 |
| T3 |  | 2.86 (1.31-6.20) | 0.008 |  | 3.33 (1.25-8.92) | 0.016 |  | 3.25 (1.41-7.51) | 0.006 |

Models adjust for: age, sex, smoking, hypertension, DM, dyslipidaemia, prior MI, prior PCI, prior stroke, multi-vessel disease, LDL-C, HDL-C, FBG, HbA1c, creatinine, UA, LVEF, statin use, and dual antiplatelet therapy.

HR, hazard ratio; CI, confidence interval; NPAR, neutrophil percentage-to-albumin ratio; CTO, chronic total occlusion; PCI, percutaneous coronary intervention.
